# Supplementary material for: Red Blood Cell Distribution Width: A Risk Factor for Prognosis in Patients with Ischemic Cardiomyopathy after Percutaneous Coronary Intervention
Source: J Clin Med. 2023 Feb 16;12(4):1584. doi: 10.3390/jcm12041584 (PMC9964585; doi:10.3390/jcm12041584)
Supplement: Supplementary file 1 [file jcm-12-01584-s001.zip › jcm-2183191-supplementary.pdf]

Supplemental information (1)

1. Ischemic HF patients were diagnosed according to the following criteria:

(1) HF diagnosis according to International Classification of Diseases (ICD) 10th revision I50.106 (left ventricular failure), I50.001 (congestive HF), I50.902 (Cardiac insufficiency), I50.919 (Diastolic heart failure HF), I50.905(Chronic HF) or I50.911 (HF, unspecified)

(2) concomitant multivessel disease (MVD) (coronary artery stenosis >50% in≥2 vessels or left main).

Supplemental information (2)

Demographics included age, and sex. Vital signs included systolic blood pressure, diastolic blood pressure, heart rate, and body mass index. Comorbidities and medical history included atrial fibrillation, hypertension, diabetes, hypercholesterolemia, anemia, thrombocytopenia, chronic kidney disease, prior stroke, prior myocardial infarction (MI), and prior percutaneous coronary intervention (PCI). Laboratory parameters included white blood cell, red blood cell, hemoglobin, platelet, creatinine, blood nitrogen urea, estimated glomerular filtration rate (eGFR), ALT (alanine transaminase), AST (aspartate transaminase), total cholesterol (TC), triglyceride (TG), low-density lipoprotein cholesterol (LDL-C), high-density lipoprotein cholesterol (HDL-C), glucose, sodium, potassium, brain natriuretic peptide (BNP) and red blood cell distribution width (RDW). Echocardiography data included left atrial diameter, left ventricular end systolic diameter (LVDs), left ventricular end diastolic diameter (LVDd) and left ventricular injection fraction (LVEF). Medication included aspirin, clopidogrel, ticagrelor, statins, calcium channel blocker (CCB), beta-blockers, angiotensin-converting enzyme inhibitor (ACEI), angiotensin receptor blocker (ARB), diuretics, sacubitril/valsartan. Angiographic data include left main artery (LM) disease and three-vessel disease, chronic total occlusion lesion (CTO), diffuse lesion, in-stent restenosis and SYNTAX score. Procedural results included target vessel territory [left anterior descending artery (LAD), left circumflex artery (LCX), right coronary artery (RCA)], complete revascularization, and number of stents.

The lesion characteristics of the coronary artery were defined as follows:

(1) LM disease: an angiographically estimated stenosis >50% or a fractional flow reserve <0.80 in the left main coronary artery ostium, mid-shaft, or distal bifurcation.

(2) three-vessel disease: more than two main coronary branches (vessel diameter ≥ 2 mm) with extent of stenosis ≥ 50%.

(3) chronic total occlusion lesion: lesion with complete obstruction [thrombolysis in myocardial infarction (TIMI) flow grade 0] lasting longer than 3 months, which was judged from the previous medical history or coronary angiogram results.
(4) diffuse lesion: a single stenotic lesion with a length of ≥ 20 mm.
(5) in-stent restenosis: stenosis of ≥ 50% occurring in the quantified by the synergy between PCI with taxus and cardiac surgery (SYNTAX) score.
(6) SYNTAX score was calculated according to the SYNTAX score algorithm (www.syntaxscore.com).
